# Belladonna for Incontinence of Urine

**Published:** 1858-04

**Authors:** 


### BELLADONNA FOR INCONTINENCE OF URINE.

A female child, nine months old, had been affected with incontinence of urine from birth. The urine continued constantly to dribble away, and the clothes of the little patient wet with this irritating secretion.

The urine was clear, slightly acid, and without any abnormal sediment. The patient commenced 10th February, 1857, 1-24 of a grain of extract of belladonna, three times a day, and gradually increased it, until March 12th, she was taking 1-12 grain, and so continued up to 26th of May, when she was so much better, that sometimes three or four nights would pass without her passing water during the whole night. Iron was now added to the belladonna, and the recovery henceforth was so complete, that the patient had complete control of the discharge; but it was necessary to continue the remedies much longer, before the irritability of the bladder was subdued, as it yet returned so soon as the belladonna was withdrawn.—*Ibid.*

